# LY2405319, an analog of fibroblast growth factor 21 ameliorates α-smooth muscle actin production through inhibition of the succinate—G-protein couple receptor 91 (GPR91) pathway in mice

**DOI:** 10.1371/journal.pone.0192146

**Published:** 2018-02-14

**Authors:** Cong Thuc Le, Giang Nguyen, So Young Park, Dae Hee Choi, Eun-Hee Cho

**Affiliations:** Department of Internal Medicine, School of Medicine, Kangwon National University, Chuncheon, Republic of Korea; National Institutes of Health, UNITED STATES

## Abstract

Fibroblast growth factor 21 (FGF21) is an important metabolic regulator expressed predominantly in the liver. In this study, we evaluated the role of LY2405319, an analogue of FGF21, in hepatic stellate cell (HSC) activation and in a methionine and choline-deficient (MCD)-diet induced mouse model of liver fibrosis. During liver injury, HSCs trans-differentiate into activated myofibroblasts which produce alpha-smooth muscle actin (α-SMA) and become a major cell type in hepatic fibrogenesis. Succinate and succinate receptor (GPR91) signaling has emerged as a regulator to promote α-SMA production in MCD diet- induced mice. Treatment with palmitate or MCD medium on LX-2 cells (HSCs) increased succinate concentration in the conditioned medium and cell lysate of LX-2 cells and increased production of GPR91 and α-SMA. However, LY2405319 administration ameliorates palmitate or MCD media-induced succinate production and decreases over-expression of GPR91 and α-SMA in LX2-cells. In an *in vivo* study, the MCD diet treatment caused increased steatohepatitis and liver fibrosis compared with the control diet in mice. Administration of LY2405319 improved steatohepatitis ameliorated GPR91 and α -SMA production in the liver, decreased succinate concentration in both liver and serum of MCD diet -induced mice. These results suggest that FGF21 reduces production of α-SMA by inhibiting the succinate-GPR91 pathway. We conclude that FGF21 acts as an inhibitor of the succinate-GPR91 pathway to control liver fibrosis. This suggests that FGF21 has therapeutic potential for treating liver fibrogenesis.

## Introduction

Hepatic fibrosis is a wound- healing response to chronic liver injury. During liver injury, activation of hepatic stellate cells (HSCs) is a major process to produce extracellular matrix structures, which eventually leads to the development of liver fibrosis [1,2].

Fibroblast growth factor 21 (FGF21) is a member of a family of growth factors known to be crucial endocrine factors and belongs to the FGF19 subfamily [3]. FGF21 has been reported to reverse hepatic steatosis and improve insulin sensitivity in high -fat diet-induced obese mice [4]. FGF21 transgenic mice fed high -fat/high -carbohydrate diet for 15 weeks showed protection against diet-induced obesity [5], and FGF21 knockout (KO) mice developed more severe steatosis and fibrosis than wild- type mice [6]. Recently, administration of FGF21 was found to ameliorate hepatic fibrogenesis in dimethylnitrosamine (DMN) -induced fibrotic livers in mice [7].

Succinate, an important intermediate of the citric acid cycle, is formed from oxidation of succinyl-CoA and converted into fumarate by succinate dehydrogenase (SDH), which is catalyzed by the enzyme succinyl-CoA hydrolase [8,9]. In hypoxic conditions or when there is a dysregulation of energy balance, succinate is secreted from the mitochondrial and acts as an extracellular signaling molecular by binding to a specific G -protein couple receptor 91 (GPR 91) [10,11]. Once GPR91 is activated, it influences several highly vascularized tissues, such as kidney, heart, retina, white adipose tissue, and liver [8,12,13].

Regarding the role of succinate in the liver, recent study reported that activated GPR91 led to stimulation of HSCs and using adeno -associated virus (AAV) -mediated RNA disruption of GPR91 gene expression in MCD diet -fed mice as a model of non -alcoholic fatty liver disease (NAFLD), considerably ameliorated steatohepatitis and fibrosis [14].

However, the relationship between FGF21 and succinate—GPR91 signaling in HSCs remains unknown. In this study, we investigated the effects of LY2405319, an FGF21 analogue, on the succinate-GPR91 pathway in HSC activation, using LX-2 cells and an MCD diet–induced mouse model of NAFLD.

## Materials and methods

### Materials

Overexpression of α-SMA was used as a standard marker for HSC activation [1]. Dulbecco’s modified Eagle’s media (DMEM), completely deficient of methionine and choline medium (MCD medium) and methionine- and choline supplements (MCS medium and control medium, respectively) were purchased from WELGENE (Kyeongsan, Korea). Palmitate and succinate were purchased from Sigma (St. Louis, MO, USA). LY2405319, a recombinant protein analogue of FGF21, was supplied by Lilly Company (Indianapolis, IN, USA).

#### 2.1. Cell culture

LX-2 cells are immortalized human hepatic stellate cells[15], and were kindly provided by Prof. Ja June Jang, Seoul National University, Korea. The LX-2 cells were cultured in DMEM with 10% fetal bovine serum supplemented with 1% penicillin/streptomycin. Cells were maintained in a humidified 37°C incubator with 5% CO_2_.

### Animals and administration of FGF21 analogue

Male C57BJ6 mice, 6-to 8 -weeks-old and weighing 22–25 g, were purchased from Doo Yeol Biotech (Seoul, Korea). All mice were housed at ambient temperature (22 ± 1°C) with a 12/12-h light/dark cycle and free access to water and food. The mice were fed with the MCD diet (MCD diet group) as an animal model of NAFLD, or control chow diet (control group) for 8 weeks. The MCD diet–group mice were randomly divided into two groups after 4 weeks of being fed the MCD diet and were administered the LY2405319, an FGF21 analogue, (1.5 mg/kg/day; n = 8) or a phosphate–buffered saline (PBS) solution (control MCD group; n = 8) daily for 4 weeks. Carbon dioxide (CO2) was used for euthanasia agent for mice. This animal research protocal has been reviewed and approved by the Institutional Animal Care and Use Committee of Kangwon university (KW-160601-1).

### Western blot analysis

Cells were lysed in radioimmunoprecipitation (RIPA) buffer containing 25 mM Tris-HCl (pH-7.6), 150 mM NaCl, 1% NP-40, 1% sodium deoxycholate, 1% SDS, and protease inhibitor mixture (Roche Diagnostics, Mannheim, Germany) on ice. Equal amounts of proteins were resolved on an SDS/PAGE gel and then electro -transferred onto PVDF membranes and blocked with 5% nonfat dry milk for 30 min at room temperature. Levels of proteins were determined by incubation with primary antibodies at appropriate dilutions. Primary antibodies included those specific for GPR91 (sc-50466, Santa Cruz Biotechnology, Santa Cruz, CA, USA), α-SMA (GTX112861, GeneTex, Irvine, CA, USA), GAPDH (GTX627408, GeneTex), AMP-activated protein kinase alpha (AMPKα, 2532, Cell Signaling Technology), pPhospho-AMPKα (Thr172) (2535, Cell Signaling Technology), collagen type 1 (COL1A1, Rockland Antibodies & Assays, Limerick, PA, USA). The membranes were further incubated with secondary antibodies conjugated to HRP, and protein bands detected using the Westsave Star Detection Reagent system (AbFrontier, Seoul, Korea). GAPDH was used as a loading control for western blot analysis.

### Succinate assay

The level of cellular succinate was determined using a succinate colorimetric assay kit (BioVision, CA, USA). Succinate levels were detected by absorbance at 450 nm with each measurement being performed in triplicate.

### Histological analysis

Samples of mouse liver were fixed in 10% (w/v) phosphate-buffered formalin for 18–20 h. After dehydration through a graded series of ethanol solutions, the tissues were embedded in paraffin wax. Serial frontal sections were cut and stained with hematoxylin and eosin (H&E), and Masson's trichrome.

### Cell viability assays and proliferation assay

Cell viability was determined by MTT assay. For MTT assay, 25,000 cells/well were seeded in 24-well plates. After 24 h of incubation, LX-2 cells were treated with Palmitate (300 μM), MCD medium and LY2405319 (100 nM) in 24h and then make assessment with MTT. MTT (50 μl, 1 mg/ml in DMEM without phenol red) was added to each well and keep for 4 h in incubator at 37°C. Absorbance was taken at 570 nm. Untreated cells at initial time of treatment were used as control (100% cell survival). For cell proliferation analysis the cell proliferation ELISA BrdU (Roche applied science) was applied.

### Statistical analyses

All data are expressed as means ± S.E. from at least three independent experiments. Data analyses for the two groups were performed by ANOVA test with p-values < 0.05 being considered statistically significant.

## Results

### LY2405319 ameliorates succinate-, palmitate- and MCD medium-induced HSCs activation

**Fig 1:**
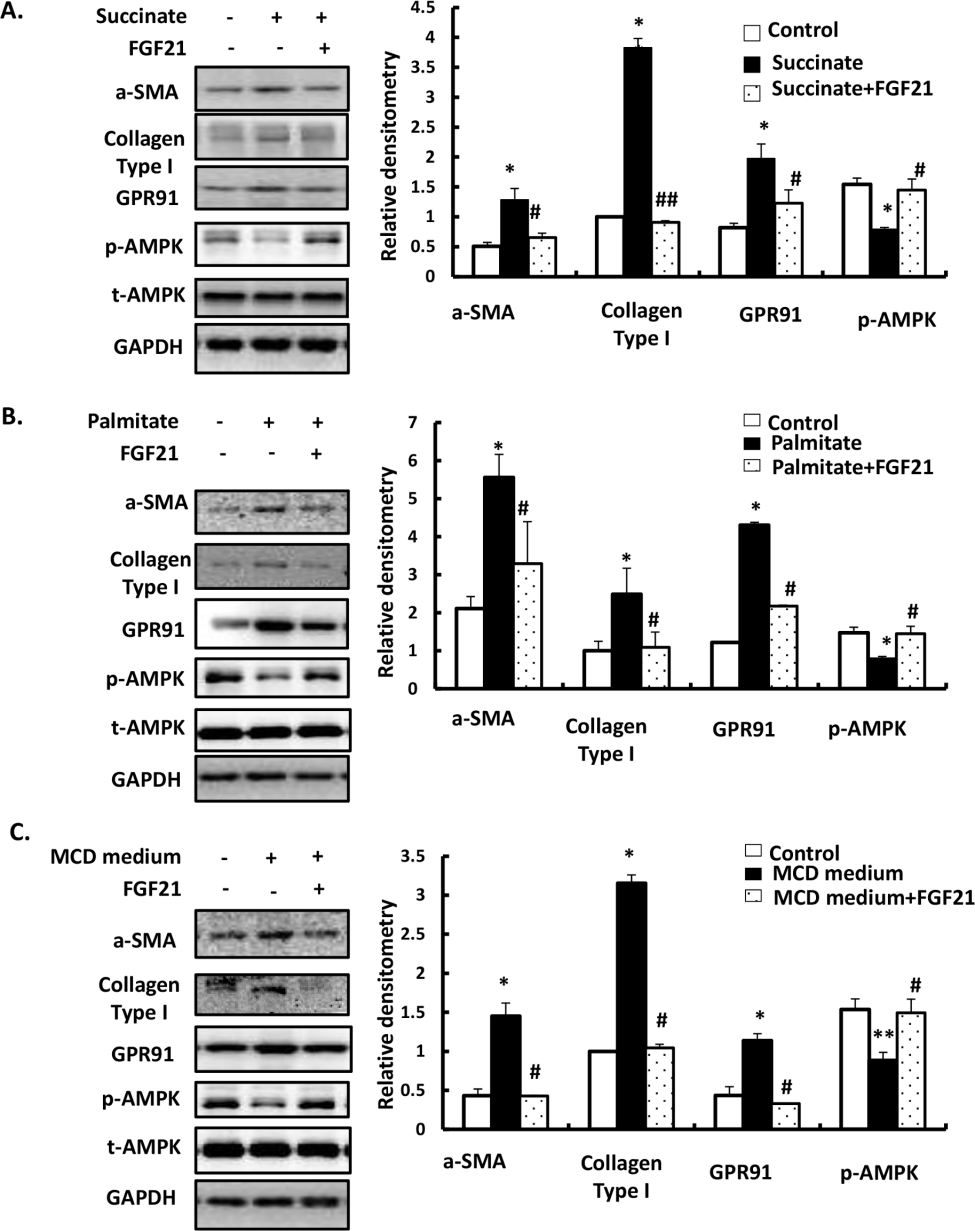
LY2405319 ameliorates α-SMA and GPR91 expression in succinate-, palmitate- and MCD media-induced activation of HSCs. Western blot analysis of α-SMA, GPR91, AMPK phosphorylation, and total AMPK were detected using specific antibodies. Densitometric analysis was performed and data are presented as the mean values ± S.E. of three independent experiments. *P < 0.05, **P < 0.01 and ***P < 0.001 significantly different from the control group. #P < 0.05, ##P < 0.01 and ###P < 0.001 significantly different from the palmitate or MCD medium. (A) LX-2 cells were treated with succinate (400 μM) and LY2405319 (100 nM) for 24 h. (B) LX-2 cells were treated with palmitate (300 μM) and LY2405319 (100 nM) for 24 h. (C) LX-2 cells were incubated with MCD medium and LY2405319 (100 nM) for 24 h.

To directly investigate the influence of succinate, palmitate and MCD medium on activation of HSCs, we cultured LX-2 cells in DMEM medium plus 10% fetal bovine serum and 1% penicillin streptomycin for 24h and then treated cell with 400 μM succinate or 300 μM palmitate or incubated with MCD medium for 24h. Western blotting was performed to evaluate α-SMA, Collagen type1 and GPR91 expressions. When LX-2 cells were incubated with succinate, palmitate and MCD medium for 24h, increase in expression of α-SMA and GPR91 was observed, which was reversed by LY2405319 (100 nM) treatment (Fig 1). Increased RNA expressions of α-SMA, Collagen type1 and GPR91 expressions induced by succinate, palmitate and MCD medium for 24h were ameliorated by LY2405319 treatment (S1 Fig). Succinate, palmitate and MCD medium decreased the phosphorylation of AMPK and LY2405319 treatment attenuated the decrease of AMPK phosphorylation. These results suggest that succinate, palmitate, and MCD medium treatment led to HSC activation, through direct activation of GPR91 and that treatment with an FGF21 analogue may block HSC activation via GPR91 downregulation or AMPK activation.

### LY2405319 attenuates succinate concentrations in extracellular media and cell lysates of LX-2 cells stimulated by palmitate or MCD treatment and inhibits cell proliferation of LX-2 cells

**Fig 2:**
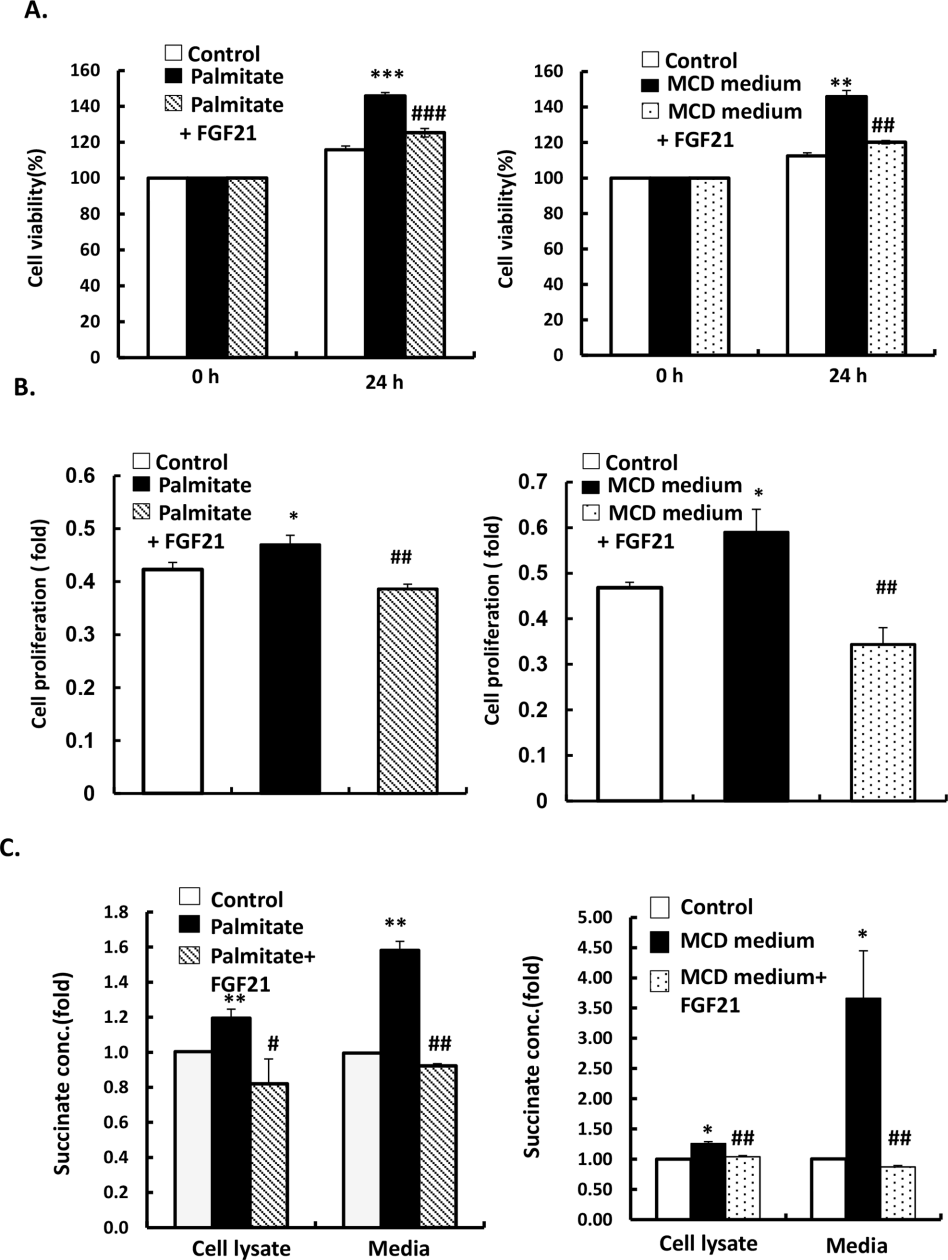
LY2405319 attenuates succinate concentration in the media and cell lysates of LX-2 upon stimulation with palmitate and MCD medium and inhibits the proliferation of LX-2 cells. LX-2 cells were exposure with palmitate (300 μM) or MCD medium for 24 h before measure cell viability by MTT assays or measure succinate concentration in whole cell lysates and media from palmitate- or MCD medium-treated LX-2 cells (n = 3). All values represented mean ± S.E. of three independent experiments. *P < 0.05, **P < 0.01 and ***P < 0.001 significantly different from the control group. #P < 0.05, ##P < 0.01 and ###P < 0.001 significantly different from the groups treated with palmitate or MCD medium.

We examined whether LY2405319 downregulates HSC activation by decreasing succinate levels and GPR91 expression in the presence of MCD medium or palmitate. The optical density of succinate concentrations in cell lysate and the media was measured. LX-2 cells exposed to palmitate (300 μM) and MCD medium for 24h showed increased secretion of succinate in their conditioned media and cell lysates. Conversely, treatment of LY2405319 (100 nM) significantly alleviated palmitate- and MCD medium- induced elevation of succinate concentration in the extracellular media and cell lysates of LX -2 cells (Fig 2C). These results suggest that palmitate and MCD medium promote succinate secretion from LX-2 cells, resulting in increased expression of GPR91 and α-SMA, which is mitigated by LY2405319 treatment.

To evaluate inhibition efficacy of LY2405319 on LX-2 cells stimulated by palmitate-or MCD medium, we cultured LX-2 cells in 24h and then administration with palmitate (300 μM) or MCD medium in next 24 h. The exposure of palmitate and MCD medium boost the growth of LX-2 cells, whereas treatment of LY2405319 (100 nM) inhibits the proliferation of LX-2 cells (Fig 2A and 2B). These results show that palmitate and MCD medium increase the proliferation of HSCs and the development of HSCs will be inhibited under the administration of FGF21 analogue.

### LY2405319 decreases expression of collagen type 1, α-SMA and GPR91 in the liver and improves steatohepatitis and fibrosis in an MCD–diet mouse model

**Fig 3:**
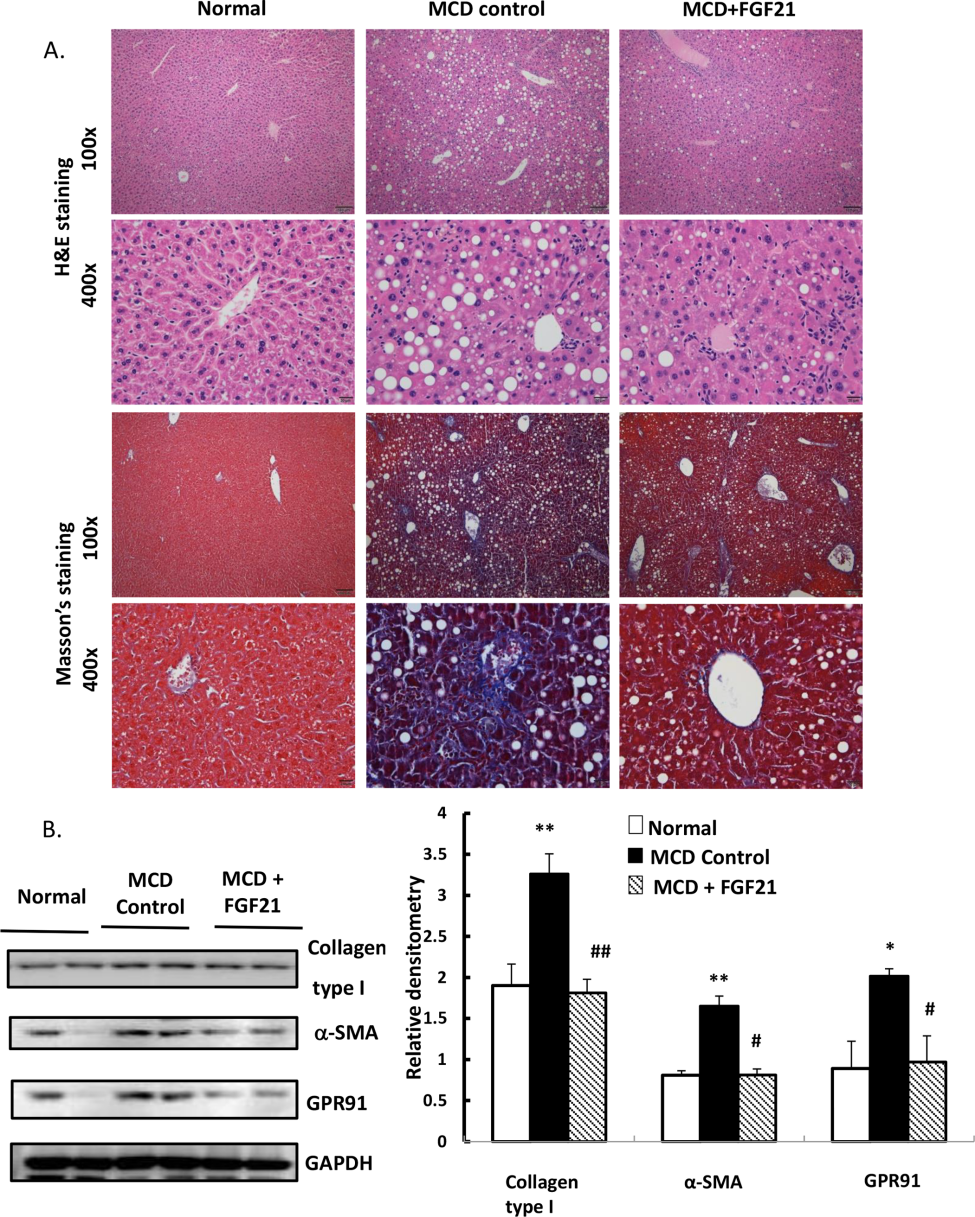
LY2405319 alters collagen type 1, α-SMA expression and phenotypes of livers from MCD diet-induced mouse models of liver fibrosis. (A) The effects of LY2405319 administration on MCD diet-induced liver fibrosis in mice. H&E staining and Masson’s trichrome. All slides are magnified 100×. The small figures of each slide are 400× magnification. (B) Western blot analysis collagen type 1, α-SMA production and GPR91 expression in livers from MCD diet-fed mice given LY2405319. Densitometric analysis was performed and data are presented as the mean values ± S.E. of four independent mice liver of each group.

A series of experimental methods were used to evaluate whether LY2405319 administration could improve hepatic fibrogenesis. The expression of α-SMA and collagen type 1 in the liver were evaluated by western blotting. The expression of α-SMA and GPR91 in the liver of mice fed with MCD diet for 8 weeks was increased compared to that in control, whereas administration of LY2405319 attenuated the increase of collagen type 1, α-SMA and GPR91 protein levels (Fig 3). Moreover, LY2405319 intraperitoneal administration for 4 weeks daily ameliorated hepatic steatosis and fibrosis that was induced by MCD diet.

### LY2405319 decreases succinate concentration in liver lysate and in the serum of MCD diet–induced mouse models of liver fibrosis

**Fig 4:**
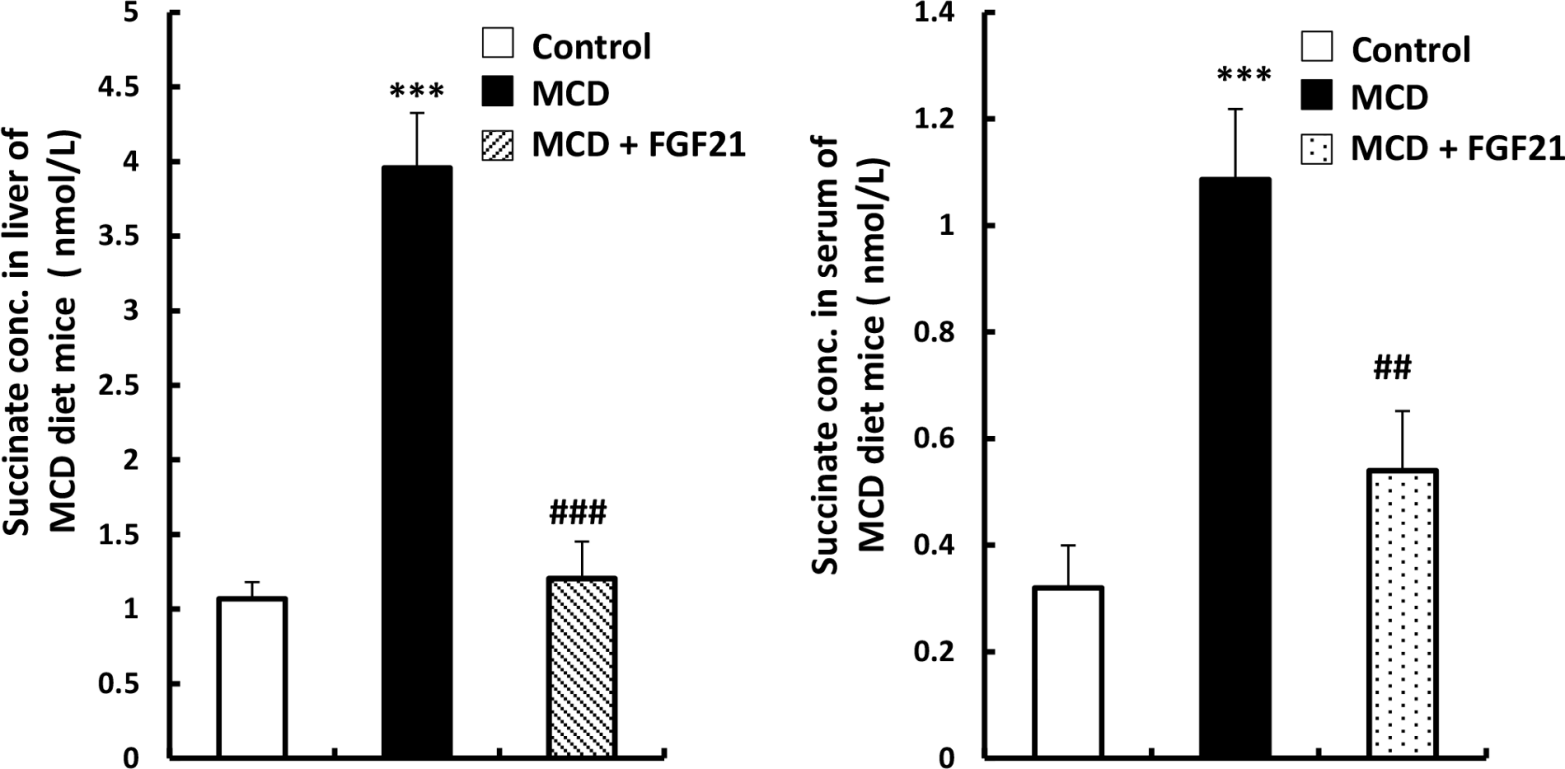
LY2405319 decreases succinate concentration in liver lysate and serum of MCD diet–induced mouse models of liver fibrosis. Measurement of succinate concentration in whole liver lysate (n = 5) and serum (n = 6) from MCD fed mice. Data are presented as the mean values ± S.E. *P < 0.05, **P < 0.01 and ***P < 0.001 significantly different from the control group. #P < 0.05, ##P < 0.01 and ###P < 0.001 significantly different from the groups of MCD diet mice.

We measured optical density of succinate concentration from liver lysate and serum to evaluate whether LY2405319 attenuates liver fibrogenesis through inhibition of succinate level. The results showed that succinate concentration in liver lysate and serum of MCD diet mice are higher than those in control groups and LY2405319 injected group (Fig 4). These results suggest that the administration of LY2405319 attenuates the increase of succinate level caused by MCD diet mice, resulting in the improvement of liver fibrogenesis stage in MCD diet mice.

## Discussion

In this study, we tested whether LY2405319, an FGF21 analogue, can be a potential therapeutic treatment for hepatic fibrosis by inhibiting HSC activation via suppressing succinate-GPR91 signaling in an MCD diet -induced mouse model of liver fibrosis. LY2405319 used as the modification of FGF21, show the same in efficacy and biological activity with native human FGF21 in cell-based, rodent, and non- human primate assay[16,17].

Succinate, known to be a TCA cycle intermediate has emerged as an important signaling molecule in ischemic liver damage or chronic liver diseases [8,14,18,19]. In a previous study, elevation in succinate concentrations in the plasma and in both isolated hepatocytes and HSCs of MCD diet -fed mice was observed compared with that in controls, suggesting both systemic and local action of succinate in HSC activation [19]. In the same study [14], increased succinate concentrations and over expression of GPR91 were found in an MCD diet- fed mouse model of NAFLD and knockout of the GPR91 gene led to attenuation of steatosis and fibrosis in an MCD diet–induced mouse model of non -alcoholic steatohepatitis.

FGF21 boosts glucose uptake, decreases hepatic gluconeogenesis, increases insulin sensitivity and lessens hyperglycemia in a diet-induced mouse model of obesity [4]. Moreover, FGF21 increases hepatic lipid oxidation and inhibits lipogenesis, thereby, causing a decline in the concentration of circulating free fatty acids and LDL-cholesterol, while increasing HDL-cholesterol levels [4]. In accordance with these observations, FGF21 has been found to prevent hepatic steatosis [4] and atherosclerosis [20] and, furthermore, it has been shown to have therapeutic potential against metabolic diseases, such as type 2 diabetes [21]. The level of FGF21 in serum has been found to be elevated in several metabolic disorders related to obesity [22]. The high level of serum FGF21 in obese subjects can be explained as a compensatory mechanism or FGF21 receptor feedback [23]. In a recent study, the hepatic expression level of β-Klotho, a critical co-receptor of FGF 21 was negatively associated with plasma FGF21 levels in mice, suggesting a resistance to FGF21 [24].

In this study, we demonstrated that both succinate concentration and GPR91 expression is increased in the fibrotic liver of MCD -induced mouse model of liver fibrosis and that the administration of LY2405319 decreased the expressions of GPR91, α-SMA and collagen type 1 in the liver of MCD fed mice. Moreover, treatment of LY2405319 inhibited the increase of succinate concentration in both liver and blood circulation of MCD diet mice. At a cellular level, treatment of LY2405319 blocked the increase in expression of GPR91 in HSCs caused by succinate. Additionally, palmitate- and MCD-supplemented medium of HSCs increased the concentrations of succinate and expressions of GPR91 and α-SMA in HSCs, which was reversed by LY2405319 co-treatment. Finally, the administration of LY2405319 inhibit proliferation of HSCs stimulated by palmitate and MCD medium. These findings suggest that LY2405319 inhibits accumulation of succinate levels and expression of its receptor GPR91, which results in blocking the growth and activation of HSCs. Therefore, the development of inhibitors or antagonists targeting GPR91 are likely to have significant therapeutic potential in the treatment of the liver fibrosis.

There are few studies on the relationship between FGF21 and hepatic stellate cell activation [7]. A recent study reported that FGF21 blocked α-SMA production of cytokine transforming growth factor-β (TGF-β)-, platelet-derived growth factor BB (PDGF-BB)-, nuclear transcription factor-κB (NF-κB)-induced HSC activation and caused apoptosis of activated HSC [7].

In the present study, we demonstrated that the expressions of GPR91, α-SMA, collagen type 1 increased in succinate-, palmitate-, and MCD -treated HSCs and this overexpression was inhibited by co-treatment with LY2405319, suggesting a novel pathway of FGF21 in suppressing the HSCs activation via GPR91 signaling.

The exploration of the molecular mechanisms by which FGF21 blocks activation of HSCs still remains an interesting area of investigation for future studies. Given that FGF21 is mainly secreted by the liver, how FGF21 controls HSC activation remains to be determined [25]. Three plausible hypotheses can be given for research direction. First, FGF21 can regulate α-SMA by activating its receptor in HSCs. Second, FGF21 can influence hepatocytes, resulting in the decrease of secretion of both succinate and inflammatory cytokines, such as TGF-β and PDGF, which play important roles in the activation of HSCs [1,14,26]. Further experiments to examine FGF21 receptor expression in HSCs and hepatocytes need to be conducted to investigate this possibility. Further, even though the main recruitment of FGF21 is from hepatocytes, its effect on the liver and other tissues possibly stem from activation of downstream signals release from other organs. It is known that FGF21 regulates metabolic processes and homeostasis of the liver by controlling adiponectin secretion from adipose tissue [27,28] and corticosterone synthesis through the hypothalamic-pituitary adrenal (HPA) axis [29,30], which can increase 5' adenosine monophosphate-activated protein kinase (AMPK) activation in the liver [28,31,32]. Interestingly, AMPK, a key metabolic player in the regulation of cellular energy homeostasis, displays clear overlap with FGF21 in metabolic profile. Both FGF21 and AMPK augment glucose uptake and reduce hyperglycemia as well as enhance free fatty acid oxidation and energy expenditure [33–35]. Additionally, it has been reported that AMPK activation decreases α-SMA -production in activated HSCs [36], suggesting that FGF21 could regulate AMPK phosphorylation to inhibit expression of α-SMA in a mouse model of NAFLD via triggering adiponectin release from adipocytes. Furthermore, whether activator or inhibitor of AMPK may influence succinate levels and GPR91 expression remains a significant unanswered question. In this study we showed that the FGF21 treatment increased AMPK phosphorylation suggesting the interaction of AMPK and FGF21. The crosstalk between the AMPK pathway and the succinate-GPR91 pathway will be crucial topic to study in the future.

The succinate—GPR91 pathway is a potential therapeutic target to ameliorate hepatic fibrogenesis in mouse models. Additionally, the efficacy of an FGF21 analogue on bile duct ligation-, and in alcohol- and virus- induced liver diseases in mice with different etiologies of liver injury has not been well elucidated. The molecular downstream mechanisms by which an FGF21 analogue mitigates hepatic fibrosis, therefore, remains to be clarified. However, the FGF21 -succinate -GPR91 signaling pathway will likely play a key role in mouse models of liver fibrosis, although it may not be exactly the same in humans, and will require further study to delineate how FGF21 and GPR91 signaling pathway may work in liver fibrosis in humans.

In clinical trials, FGF21 analogues including LY2405319 produced by Eli Lilly Company and PF05231023 (PF) produced by Pfizer, induced a dose -dependent reduction in levels of plasma triglycerides, low-density lipoprotein cholesterol, and total cholesterol, and an elevation in levels of high-density lipoprotein cholesterol compared with placebo treatment [21,37]. However, the glycemic endpoint in humans was not significantly altered in these studies [21,37]. The overall results from this clinical experiment were positive and indicative of the translational relevance of FGF21 pharmacology from animals to humans.

In conclusion, this research demonstrated that administration of FGF21 reduces the production of α-SMA through inhibition of succinate -GPR91 signaling in HSCs and improves hepatic steatosis and fibrosis in an MCD diet -induce mouse model. Owing to the therapeutic potential of FGF21 analogue and inhibition of succinate signaling, the development of FGF21 agonists and drug delivery systems targeting both FGF21 and the succinate -GPR91 pathway in humans requires further investigation.

## Supporting information

S1 Fig Increased RNA expressions of α-SMA, Collagen type1 and GPR91 expressions induced by succinate, palmitate and MCD medium for 24h were ameliorated by LY2405319 treatment. Data are presented as the mean values ± S.E. of three independent experiments. *P < 0.05, **P < 0.01 and ***P < 0.001 significantly different from the control group. #P < 0.05, ##P < 0.01 and ###P < 0.001 significantly different from the palmitate or MCD medium. (A) LX-2 cells were treated with palmitate (300 uM) and LY2405319 (100 nM) for 24 h. (B) LX-2 cells were treated with MCD medium and LY2405319 (100 nM) for 24 h. (C) LX-2 cells were incubated with succinate (400 uM) and LY2405319 (100 nM) for 24 h.
(TIF)

## References

[1] Friedman SL. Mechanisms of hepatic fibrogenesis. Gastroenterology. 2008 134:1655–69. doi: 10.1053/j.gastro.2008.03.003 18471545 10.1053/j.gastro.2008.03.003 PMC2888539

[2] Kisseleva T, Brenner DA. Hepatic stellate cells and the reversal of fibrosis. Gastroenterol Hepatol. 2006;21(Suppl. 3):S84–S7. 10.1111/j.1440-1746.2006.04584.x 16958681

[3] Ornitz DM, Itoh N. The Fibroblast Growth Factor signaling pathway. Wiley Interdiscip Rev Dev Biol. 2015 May-Jun;4(3):215–66. doi: 10.1002/wdev.176 25772309 10.1002/wdev.176 PMC4393358

[4] Xu J, Lloyd DJ, Hale C, Stanislaus S, Chen M, Sivits G, et al Fibroblast growth factor 21 reverses hepatic steatosis, increases energy expenditure, and improves insulin sensitivity in diet-induced obese mice. Diabetes 2009;58:250–9. doi: 10.2337/db08-0392 18840786 10.2337/db08-0392 PMC2606881

[5] Kharitonenkov A, Shiyanova TL, Koester A, Ford A, Micanovic R, Galbreath EJ. FGF-21 as a novel metabolic regulator. J Clin Invest. 2005 115(6):1627–35. doi: 10.1172/JCI23606 15902306 10.1172/JCI23606 PMC1088017

[6] Fisher FM, Chui PC, Nasser IA, Popov Y, Cunniff JC, Lundasen T. Fibroblast growth factor 21 limits lipotoxicity by promoting hepatic fatty acid activation in mice on methionine and choline-deficient diets. Gastroenterology. 2014 147(5):1073–83. doi: 10.1053/j.gastro.2014.07.044 25083607 10.1053/j.gastro.2014.07.044 PMC4570569

[7] Xu P, Zhang Y, Liu Y, Yuan Q, Song L, Liu M. Fibroblast growth factor 21 attenuates hepatic fibrogenesis through TGF-β/smad2/3 and NF-κB signaling pathways. Toxicol Appl Pharmacol. 2016;290:43–53. doi: 10.1016/j.taap.2015.11.012 26592322 10.1016/j.taap.2015.11.012

[8] Ariza AC, Deen PM, Robben JH. The succinate receptor as a novel therapeutic target for oxidative and metabolic stress-related conditions. Front-Endocrinol. 2012;Lausanne(3): 22. 10.3389/fendo.2012.00022 PMC3355999 22649411

[9] Krebs HA. Rate control of the tricarboxylic acid cycle. Adv Enzyme Regul 1970;8:335–53 4920378 10.1016/0065-2571(70)90028-2

[10] He W, Miao FJ, Lin DC, Schwandner RT, Wang Z, Gao J, et al Citric acid cycle intermediates as ligands for orphan G-protein-coupled receptors. Nature. 2004;429 188–93. doi: 10.1038/nature02488 15141213 10.1038/nature02488

[11] Hebert SC. Physiology: orphan detectors of metabolism. Nature. 2004; 429:143–5. doi: 10.1038/429143a 15141197 10.1038/429143a

[12] Regard JB, Sato IT, Coughlin SR. Anatomical profiling of G protein-coupled receptor expression. Cell 2008;135: 561–71. doi: 10.1016/j.cell.2008.08.040 18984166 10.1016/j.cell.2008.08.040 PMC2590943

[13] McCreath KJ, Espada S, Galvez BG, Benito M, Molina DA, Sepulveda P, et al Targeted disruption of the SUCNR1 metabolic receptor leads to dichotomous effects on obesity. Diabetes. 2015;64:1154–67. doi: 10.2337/db14-0346 25352636 10.2337/db14-0346

[14] Li YH, Choi DH, EH L, Seo SR, Lee SK, Cho EH. SIRT3 regulates α-SMA production through the succinate dehydrogenase GPR91 pathway in hepatic stellate cells. J Biol Chem. 2016;291:10277–92. doi: 10.1074/jbc.M115.692244 26912655 10.1074/jbc.M115.692244 PMC4858976

[15] Xu L, Hui AY, Albanis E, Arthur MJ, O'Byrne SM, Friedman SL. Human hepatic stellate cell lines, LX-1 and LX-2: new tools for analysis of hepatic fibrosis. Gut. 2005 1;54(1):142–51. doi: 10.1136/gut.2004.042127 15591520 10.1136/gut.2004.042127 PMC1774377

[16] Kharitonenkov A, John MB, Radmila M, Thomas FB, David EM. Rational Design of a Fibroblast Growth Factor 21-Based Clinical Candidate, LY2405319. PLoS ONE 2013;8(3):e58575 doi: 10.1371/journal.pone.0058575 23536797 10.1371/journal.pone.0058575 PMC3594191

[17] Adams Carolyn A, Halstead Kharitonenkov A. LY2405319, an engineered FGF21 variant, improves the metabolic status of diabetic monkeys. PLoS ONE 2013;8:e65763 doi: 10.1371/journal.pone.0065763 23823755 10.1371/journal.pone.0065763 PMC3688819

[18] Correa PR, Kruglov EA, Thompson M, Leite MF, Dranoff JA, Nathanson MH. Succinate is a paracrine signal for liver damage. J Hepatology 2007;47 262–9. 10.1016/j.jhep.2007.03.016 PMC1986575 17451837

[19] Li YH, Woo SH, Choi DH, Cho EH. Succinate causes alpha-SMA production through GPR91 activation in hepatic stellate cells. Biochem Biophys Res Commun. 2015; 463: 853–8. doi: 10.1016/j.bbrc.2015.06.023 26051274 10.1016/j.bbrc.2015.06.023

[20] Jin L, Lin Z, Xu A. Fibroblast growth factor 21 protects against atherosclerosis via fine-tuning the multiorgan crosstalk. Diabetes Metab J. 2016; 40:22–31. doi: 10.4093/dmj.2016.40.1.22 26912152 10.4093/dmj.2016.40.1.22 PMC4768047

[21] Gaich G, Chien JY, Fu H, Glass LC, Deeg MA, Holland WL. The effects of LY2405319, an FGF21 analog, in obese human subjects with type 2 diabetes. Cell Metab. 2013;18:333–40. doi: 10.1016/j.cmet.2013.08.005 24011069 10.1016/j.cmet.2013.08.005

[22] Zhang X, Yeung DC, Karpisek M, Stejskal D, Zhou ZG, Liu F. Serum FGF21 levels are increased in obesity and are independently associated with the metabolic syndrome in humans. Diabetes. 2008;57:1246–53. doi: 10.2337/db07-1476 18252893 10.2337/db07-1476

[23] Fisher FM, Chui PC, Antonellis PJ, Bina HA, Kharitonenkov A, Flier JS. Obesity is a fibroblast growth factor 21 (FGF21)-resistant state. Diabetes. 2010;59:2781–9. doi: 10.2337/db10-0193 20682689 10.2337/db10-0193 PMC2963536

[24] Rusli F, Deelen J, Andriyani E, Boekschoten MV, Lute C, Van den Akker EB. Fibroblast growth factor 21 reflects liver fat accumulation and dysregulation of signalling pathways in the liver of C57BL/6J mice. Sci Rep. 6(30484):2016 doi: 10.1038/srep30484 27470139 10.1038/srep30484 PMC4965761

[25] Markan KR, Naber MC, Ameka MK, Anderegg MD, Mangelsdorf DJ, Kliewer SA. Circulating FGF21 is liver derived and enhances glucose uptake during refeeding and overfeeding. Diabetes. 2014; 63:4057–63. doi: 10.2337/db14-0595 25008183 10.2337/db14-0595 PMC4238010

[26] Friedman SL. The cellular basis of hepatic fibrosis. New Engl J Med. 1993;328:1828–35. doi: 10.1056/NEJM199306243282508 8502273 10.1056/NEJM199306243282508

[27] Lin Z, Tian H, Lam KS, Lin S, Hoo RC, Konishi M. Adiponectin mediates the metabolic effects of FGF21 on glucose homeostasis and insulin sensitivity in mice. Cell Metab. 2013;17:779–89. doi: 10.1016/j.cmet.2013.04.005 23663741 10.1016/j.cmet.2013.04.005

[28] Combs TP, Marliss EB. Adiponectin signaling in the liver. Rev Endocr Metab Disord. 2014;15:137–47. doi: 10.1007/s11154-013-9280-6 24297186 10.1007/s11154-013-9280-6 PMC4152934

[29] Liang Q, Zhong L, Zhang J, Wang Y, Bornstein SR, Triggle CR. FGF21 maintains glucose homeostasis by mediating the cross talk between liver and brain during prolonged fasting. Diabetes. 2014; 63:4064–75. doi: 10.2337/db14-0541 25024372 10.2337/db14-0541

[30] Owen BM, Ding X, Morgan DA, Coate KC, Bookout AL, Rahmouni K. FGF21 acts centrally to induce sympathetic nerve activity, energy expenditure, and weight loss. Cell Metab. 2014; 20:670–7. doi: 10.1016/j.cmet.2014.07.012 25130400 10.1016/j.cmet.2014.07.012 PMC4192037

[31] Yamauchi T, Kamon J, Minokoshi Y, Ito Y, Waki H, Uchida S. Adiponectin stimulates glucose utilization and fatty-acid oxidation by activating AMP-activated protein kinase. Nat Med. 2002;8:1288–95. doi: 10.1038/nm788 12368907 10.1038/nm788

[32] Christ-Crain M, Kola B, Lolli F, Fekete C, Seboek D, Wittmann G. AMP-activated protein kinase mediates glucocorticoid-induced metabolic changes: a novel mechanism in Cushing's syndrome. FASEB J 2008;22:1672–83. doi: 10.1096/fj.07-094144 18198220 10.1096/fj.07-094144

[33] Lage R, Dieguez C, Vidal-Puig A, Lopez M. AMPK: a metabolic gauge regulating whole-body energy homeostasis. Trends Mol Med. 2008;14:539–49. doi: 10.1016/j.molmed.2008.09.007 18977694 10.1016/j.molmed.2008.09.007

[34] Woo YC, Xu A, Wang Y, Lam KS. Fibroblast growth factor 21 as an emerging metabolic regulator: clinical perspectives. ClinEndocrinol (Oxf) 2013;78:489–96. 10.1111/cen.12095 23134073

[35] Giralt M, Gavalda-Navarro A, Villarroya F. Fibroblast growth factor-21, energy balance and obesity. Mol Cell Endocrinol 2015;418:66–73. doi: 10.1016/j.mce.2015.09.018 26415590 10.1016/j.mce.2015.09.018

[36] Adachi M, Brenner DA. High molecular weight adiponectin inhibits proliferation of hepatic stellate cells via activation of adenosine monophosphate-activated protein kinase. Hepatology. 2008;47:677–85. doi: 10.1002/hep.21991 18220291 10.1002/hep.21991

[37] Dong JQ, Rossulek M, Somayaji VR, Baltrukonis D, Liang Y, Hudson K. Pharmacokinetics and pharmacodynamics of PF-05231023, a novel long-acting FGF21 mimetic, in a first-in-human study. Br J Clin Pharmacol. 2015;80(5):1051–63. doi: 10.1111/bcp.12676 25940675 10.1111/bcp.12676 PMC4631178

