# Supplementary figures and images for: LY2405319, an analog of fibroblast growth factor 21 ameliorates α-smooth muscle actin production through inhibition of the succinate—G-protein couple receptor 91 (GPR91) pathway in mice
Source: PLoS One. 2018 Feb 14;13(2):e0192146. doi: 10.1371/journal.pone.0192146 (PMC5812602; doi:10.1371/journal.pone.0192146)

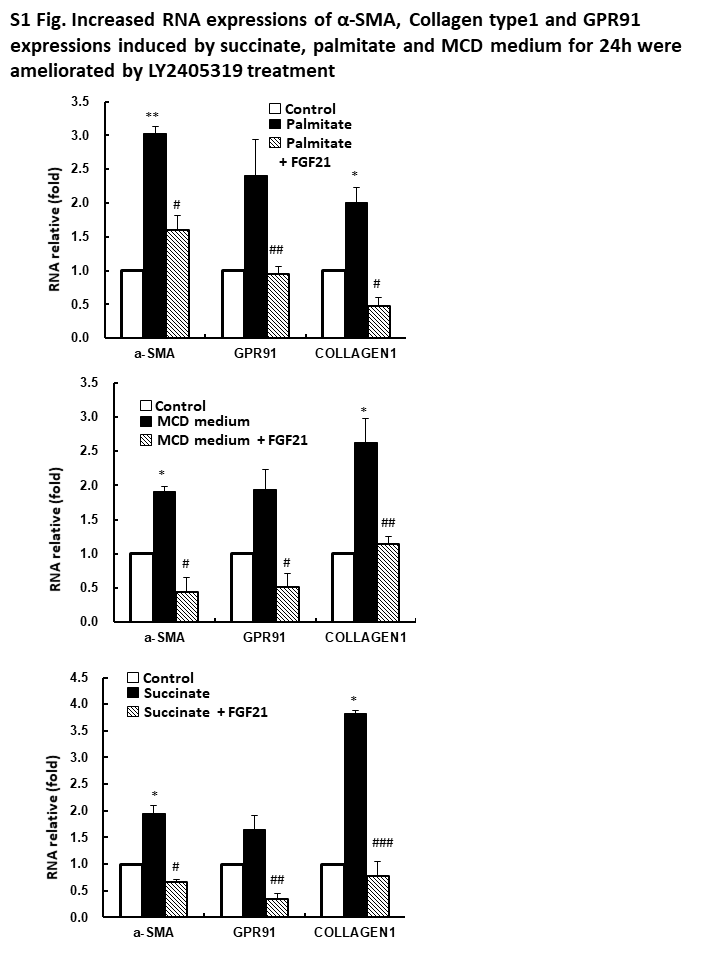

Supplement: S1 Fig — Data are presented as the mean values ± S.E. of three independent experiments. *P < 0.05, **P < 0.01 and ***P < 0.001 significantly different from the control group. #P < 0.05, ##P < 0.01 and ###P < 0.001 significantly different from the palmitate or MCD medium. (A) LX-2 cells were treated with palmitate (300 uM) and LY2405319 (100 nM) for 24 h. (B) LX-2 cells were treated with MCD medium and LY2405319 (100 nM) for 24 h. (C) LX-2 cells were incubated with succinate (400 uM) and LY2405319 (100 nM) for 24 h. (TIF) [file pone.0192146.s001.tif]
